# Transformation of Feature Selectivity From Membrane Potential to Spikes in the Mouse Superior Colliculus

**DOI:** 10.3389/fncel.2018.00163

**Published:** 2018-06-19

**Authors:** Xuefeng Shi, Yanjiao Jin, Jianhua Cang

**Affiliations:** ^1^Department of Neurobiology, Northwestern University, Evanston, IL, United States; ^2^Tianjin Eye Hospital, Tianjin Key Laboratory of Ophthalmology and Visual Science, Tianjin Eye Institute, Clinical College of Ophthalmology, Tianjin Medical University, Tianjin, China; ^3^General Hospital, Tianjin Medical University, Tianjin, China; ^4^Department of Biology and Department of Psychology, University of Virginia, Charlottesville, VA, United States

**Keywords:** superior colliculus, feature selectivity, receptive field, membrane potential, spike rate

## Abstract

Neurons in the visual system display varying degrees of selectivity for stimulus features such as orientation and direction. Such feature selectivity is generated and processed by intricate circuit and synaptic mechanisms. A key factor in this process is the input-output transformation from membrane potential (V_m_) to spikes in individual neurons. Here, we use *in vivo* whole-cell recording to study V_m_-to-spike transformation of visual feature selectivity in the superficial neurons of the mouse superior colliculus (SC). As expected from the spike threshold effect, direction and orientation selectivity increase from V_m_ to spike responses. The degree of this increase is highly variable, and interestingly, it is correlated with the receptive field size of the recorded neurons. We find that the relationships between V_m_ and spike rate and between V_m_ dynamics and spike initiation are also correlated with receptive field size, which likely contribute to the observed input-output transformation of feature selectivity. Together, our findings provide useful information for understanding information processing and visual transformation in the mouse SC.

## Introduction

Each neuron functions as an information processing unit that transforms synaptic input to spiking output. How individual neurons convert membrane potential (V_m_) dynamics to spiking activity in this transformation process is a key factor in determining how they encode sensory stimuli or motor commands. Several biophysical factors underlying V_m_-to-spike transformation have been revealed to contribute to response selectivity in the visual cortex, where neurons are tuned to features such as stimulus orientation and motion direction (Hubel and Wiesel, 1959, 1962; Ferster and Miller, 2000; Priebe, 2016). For example, spiking threshold enhances feature selectivity of the spike response over that of the membrane potential (Carandini and Ferster, 2000; Priebe and Ferster, 2008, 2012; Tan et al., 2011). Interestingly, spiking threshold is not a fixed value, but depends on the rate of V_m_ depolarization and firing history (Azouz and Gray, 2000; Wester and Contreras, 2013; Fontaine et al., 2014). In addition, trial-to-trial response variability and dendritic processing are also known to play important roles in enhancing feature selectivity of neuronal output in visual cortex (Anderson et al., 2000; Miller and Troyer, 2002; Carandini, 2007; Finn et al., 2007; Smith et al., 2013).

In recent years, the mouse superior colliculus (SC) has become a productive model for investigating neural processing in the brain (Cang et al., 2018; Ito and Feldheim, 2018). Neurons in its superficial layers, including the stratum griseum superficiale (SGS) and stratum opticum (SO), display diverse visual feature selectivity (Wang et al., 2010; Gale and Murphy, 2014; Inayat et al., 2015). Importantly, even though direction and orientation selectivity are seen in the mouse SC, their underlying circuit and synaptic mechanisms are likely different from those in the visual cortex. Orientation and direction selectivity in cat V1 arise from the precise alignment of non-selective thalamic receptive fields (RFs; Ferster and Miller, 2000; Lampl et al., 2001; Priebe and Ferster, 2005, 2008). A similar feedforward model seems to also underlie orientation selectivity in layer 4 of mouse V1 (Li et al., 2013; Lien and Scanziani, 2013), even though many of the thalamic inputs are already selective (Marshel et al., 2012; Piscopo et al., 2013; Scholl et al., 2013; Zhao et al., 2013a; Sun et al., 2016). In contrast, in the mouse SC, we recently revealed that the direction selectivity (DS) in the SC originates from the direction selective ganglion cells in the retina (Shi et al., 2017). In addition, another prominent characteristic of the SC is that it contains a much higher proportion of inhibitory neurons than in cortex (Mize, 1988, 1992; Endo et al., 2003; Inayat et al., 2015). Together, these important differences between visual cortex and the SC argue for the need to study V_m_-to-spike transformation in the mouse SC and how it contributes to feature selectivity.

In this study, we set out to describe the feature selectivity transformation from V_m_ to spikes in mouse SGS neurons and to reveal underlying contributing factors. With *in vivo* whole-cell recording, we show that the transformation of direction and orientation selectivity from V_m_ to spikes is highly variable in the mouse SGS. Interestingly, the degree of this variable transformation is correlated with RF size. Several biophysical factors, including V_m_-to-spike-rate relationship and the V_m_-rising slope for spike initiation, but not dendritic processing, are correlated with RF size and likely contribute to the observed input-output transformation of feature selectivity. Together, these findings provide useful information for understanding information processing and visual transformation in the SC.

## Materials and Methods

### Animal Preparation

C57BL/6 wild type (*n* = 23) and transgenic mice (*n* = 18) of both sexes between postnatal day 45 and 90 were used in this study. *Gad2-IRES-cre* (Stock no. 010802) and Ai32 (*RCL-ChR2(H134R)/EYFP*, Stock no. 012569) mice were acquired from Jackson Laboratory and crossed to generate heterozygous offspring that express ChR2 in glutamate decarboxylase 2 positive (GAD2^+^) cells for *in vivo* optogenetic experiments. All mice were kept on a 12 h light:12 h dark cycle, 1–5 mice per cage. All experimental procedures were approved by the Northwestern University Institutional Animal Care and Use Committee.

Mice were anesthetized with urethane (1.2 g/kg in 10% saline solution, i.p.) and then sedated by chlorprothixene (10 mg/kg in water, i.m.) as described before (Zhao et al., 2014; Shi et al., 2017). Atropine (0.3 mg/kg in 10% saline) and dexamethasone (2 mg/kg in 10% saline) were administrated subcutaneously. The animal’s body temperature was monitored through a rectal thermos-probe and maintained at 37°C through a feedback heater control module (Frederick Haer Company, Bowdoinham, ME, USA). Additional urethane (0.2–0.3 g/kg) was administered when necessary according to the depth of anesthesia monitored with toe-pinch reflex test during experiments. After the mice were anesthetized, the scalp was shaved and skin removed to expose the skull. A metal plate was mounted on top of the skull with Metabond (Parkell, Edgewood, NY, USA) mixed with black ink. The plate was then mounted to a steel stand on the vibration isolation table. A thin layer of silicon oil was applied on both eyes to prevent from drying. A craniotomy (~4.0 × 2.0 mm^2^) was performed on the left hemisphere, and the tissues including the entire V1 overlaying the SC was removed by aspiration to expose the SC.

### *In Vivo* Whole-Cell Recording

Blind whole-cell patch clamp was performed to record SGS neurons intracellularly, following procedures previously described (Shi et al., 2017). K^+^-based internal solution used in this study contained 135 mM K-gluconate, 7 mM KCl, 0.5 mM EGTA, 10 mM HEPES, 10 mM Na-phosphocreatine, 4 mM Mg-ATP, 0.4 mM Na-GTP and 0.5% biocytin with pH adjusted to 7.25. Glass pipettes were advanced perpendicularly to the horizontal plane of the mouse head until just touching the SC surface. 2% agarose in artificial cerebrospinal fluid solution (ACSF, containing 140 mM NaCl, 2.5 mM KCl, 11 mM Glucose, 20 mM HEPES, 2.5 mM CaCl_2_, 3 mM MgSO_4_, 1 mM NaH_2_PO_4_) was then added onto the exposed SC. Pipettes were then inserted into the SC.

Electrical signals were amplified using MultiClamp 700B (Axon Instruments, CA, USA), and acquired with System 3 workstation (Tucker Davis Technologies, Alachua, FL, USA) at 10 kHz. Pipette capacitance and the electrode resistance were compensated initially. Only responsive cells with stable resting membrane potentials and series resistances lower than 80 MΩ across the duration of the recordings were included in this study. The depths of recorded cells (reading from the micromanipulator) were between 0 μm and 300 μm from the point where the pipette broke into the thin membrane on the SC.

After recordings, mice were euthanized with 50 mg/kg pentobarbital and perfused with PBS and then 4% paraformaldehyde (PFA). The brain was immersed in 4% PFA overnight. Coronal slices of 150 μm were cut from the fixed brain using a vibrating blade microtome (VT1000S, Leica Microsystems). The labeled cells were revealed by visualizing biocytin with streptavidin-Alex Fluor 488 conjugate (Invitrogen). Images were captured using a Zeiss LSM5 Pascal confocal microscope (Carl Zeiss, Jena, Germany).

### Visual Stimulation

Visual stimuli were generated with Matlab Psychophysics toolbox on a CRT monitor (40 cm × 30 cm, 60 Hz, ~35 cd/m^2^ luminance). The monitor was placed 25 cm away from the right eye (contralateral to the recording site), and slightly adjusted for each cell so that its RF was completely covered. The left eye was covered throughout the experiments. Sweeping white bars on a gray background, 5° wide drifting at a speed of 30°/s, were used in the experiments to determine the direction selectivity or orientation selectivity. The drifting directions were varied between 0° and 330° (12 steps, 30° spacing), which were presented in a pseudorandom sequence together with a “blank stimulus” (gray screen at the mean luminance). The inter-stimulus interval was 0.5 or 1 s. Each stimulus was repeated 5–8 times.

### Optogenetic Determination of Cell Types

An optic fiber (0.2 mm core diameter) driven by a blue LED (470 nm, Doric Lenses) was placed ~0.5 mm above the exposed SC to photostimulate ChR2-expressing cells. The intensity of LED light was ~160 mW/mm^2^ at the tip of the optic fiber in all recordings, which was confirmed to be reliably effective in activating ChR2-expressing neurons (Shi et al., 2017).

### Data Analysis

Whole-cell recording data were first analyzed using a custom MATLAB program (Shi et al., 2017). Briefly, spikes were detected when the first derivative of raw voltage traces (dV/dt) reached a manually set positive threshold. Individual traces were carefully inspected to ensure proper spike detection. To precisely determine the start of a spike (spike initiation), the second derivative of raw voltage traces (d^2^V/dt^2^) were calculated. The spike initiation time point was the time of the first peak of d^2^V/dt^2^ of the spike (Platkiewicz and Brette, 2010). The peri-stimulus spike time histograms (PSTHs) were calculated by trial-averaging the spike counts in each 50 ms time bin. Subthreshold V_m_ were extracted by removing spikes from the raw voltage traces by a 6 ms median filter. The subthreshold V_m_ traces were trial-averaged for each stimulus condition. The trial-averaged V_m_ trace for the blank stimulus (i.e., gray screen) was used to calculate the V_m_ baseline and the standard deviation of spontaneous V_m_ fluctuations. The V_m_ baseline was then subtracted from the trial-averaged V_m_ trace for each visual stimulus condition.

For analyzing V_m_, we determined time windows of responses to the sweeping bars as described in a previous article (Shi et al., 2017). Briefly, we first calculated a cutoff threshold, which was the V_m_ level 2 standard deviations away from the baseline fluctuation. The widest segment of the traces that were above the threshold was determined as the response time window for each stimulus condition. This time window was expanded if there were any short above-threshold segments within 150 ms of the two sides. Next, the conditions that evoked wider time windows were used to guide the analysis of other conditions, to ensure that the estimation of response window was not too conservative or inaccurate for non-preferred directions. Specifically, for conditions where the window was narrower than 1/3 of the widest window of this cell (or 333 ms if the widest window is bigger than 1 s), the response time window determined from the opposite direction, reversed in timing, was used. All traces were checked visually to confirm that the time windows were determined properly. Peak V_m_ and spike rate were calculated for the response time window of each stimulus condition, subtracting the mean values of blank condition. The RF size was determined using the widest time window of all the 12 stimulus conditions, i.e., the size of the long axis of the RF. We also calculated the area of RF using a fitting method and observed similar correlations as using the width of the long axis. These analyses were thus not presented in the article.

To quantify the degree of direction selectivity and orientation selectivity, we calculated a global direction selectivity index (gDSI) and a global orientation selectivity index (gOSI). gDSI or gOSI is the vector sum of responses normalized by the scalar sum of responses in 12 directions or six orientations (Mazurek et al., 2014; Inayat et al., 2015; Shi et al., 2017): $gDSI=\left|\frac{\sum R_{\theta }e^{i\theta }}{\sum R_{\theta }}\right|$, $gOSI=\left|\frac{\sum R_{\theta }e^{i2\theta }}{\sum R_{\theta }}\right|$, where *R_θ_* is the response magnitude of spikes or V_m_, at θ direction of bars. DS transformation index (DSTI) is the difference of gDSI of spike rate and V_m_: DSTI = gDSI-Spike − gDSI-V_m_. OS transformation index (OSTI) is the difference of gOSI of spike rate and V_m_: OSTI = gOSI-Spike − gOSI-V_m_.

To quantify the relationship between V_m_ and spike rate, we fitted the data with a power law function (Hansel and van Vreeswijk, 2002; Miller and Troyer, 2002; Priebe et al., 2004): *R*(*V*_m_) = *k*(*V*_m_ − *V*_rest_)^p^, where R(V_m_) is the average spike rate at V_m_, k is a gain factor, V_rest_ is the resting membrane potential, and p is the exponent of the fitted function, referred to as power-law index in this study.

### Statistics

All pooled data were presented as mean ± SEM unless otherwise stated. Statistical significance was calculated using two-sided student’s *t* test or non-parametric, two-sided, Kolmogorov-Smirnov (K-S) test as mentioned in the text. All analyses and graph plotting were performed in MATLAB (MathWorks) or Prism (GraphPad Software Inc). No statistical methods were used to predetermine sample sizes, but our sample sizes are similar to those reported in the field.

## Results

### Feature Selectivity Transformation From V_m_ to Spike Rate in SGS Neurons

**Figure 1:**
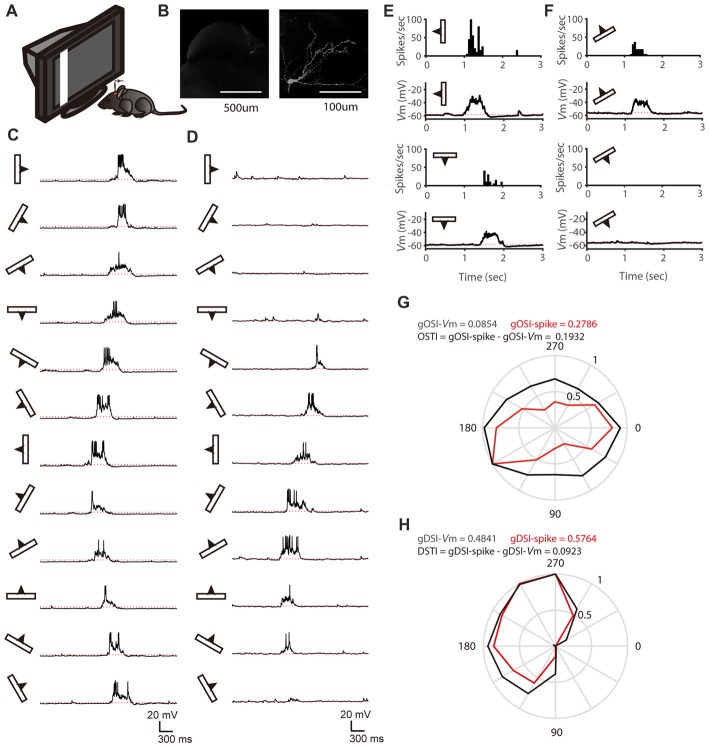
*In vivo* whole-cell recording of stratum griseum superficiale (SGS) neurons. **(A)** A schematic of the experimental setup. **(B)** Morphology of an example SGS neuron with low (left) and high (right) magnification. **(C,D)** Membrane potential (V_m_) traces of an orientation selectivity (OS) cell **(C)** and a direction selectivity (DS; **D)** cell in response to sweeping bars. The movement direction is diagramed by the bar and arrow to the left of each trace. Action potentials are truncated at −10 mV to better reveal visually-evoked V_m_ responses. The red dotted lines indicate the resting membrane potential of −58 mV in the OS cell and −56 mV in the DS cell. **(E)** The mean spike and V_m_ responses to bars moving along its preferred and opposite orientations of the same cell in **(C)**. Peri-stimulus spike time histograms (top) and trial-averaged V_m_ (bottom) are shown. The red dotted lines indicate the resting V_m_. **(F)** The mean spike and V_m_ responses to bars moving along its preferred and opposite directions of the same cell in **(D)**. **(G)** Orientation tuning for peak V_m_ (black) and spike rate (red) of the same cell in **(C)**. **(H)** Direction tuning for peak V_m_ (black) and spike rate (red) of the same cell in **(D)**.

**Figure 2:**
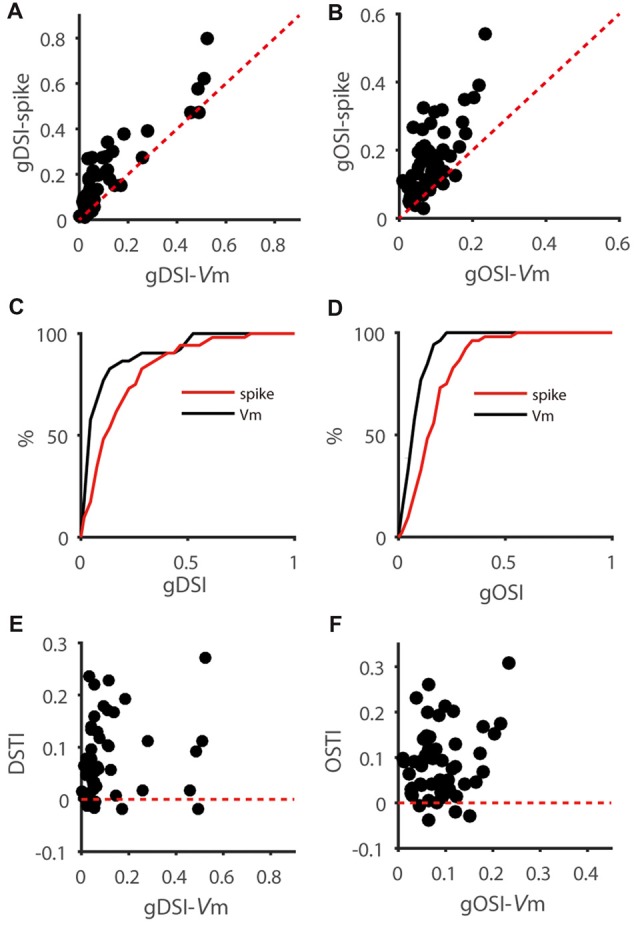
Feature selectivity transformation from V_m_ to spikes is highly variable in SGS neurons.** (A)** Scatter plot of global direction selectivity index (gDSI)-Spike vs. gDSI-V_m_ (*n* = 52 cells from 41 mice). The red dotted line is the line of unity. Note that this plot is adapted from Figure 1F of Shi et al., 2017. **(B)** Scatter plot of global orientation selectivity index (gOSI)-Spike vs. gOSI-V_m_ of the same neurons. The red dotted line is the line of unity. **(C,D)** Cumulative distributions of gDSI **(C)** and gOSI **(D)** for V_m_ and spike rate, indicating that spike rate has higher gDSI and gOSI values than V_m_ (*P* < 0.0001 for gDSI and <0.0001 for gOSI, K-S statistic = 0.46 for gDSI and 0.5 for gOSI, Kolmogorov-Smirnov test; *n* = 52 cells from 41 mice). **(E)** Scatter plot of gDSI for V_m_ vs. DS transformation index (DSTI). The red dotted line is the line of zero. Note that the DSTI is highly variable for different degree of gDSI. **(F)** Scatter plot of gOSI for V_m_ vs. OS transformation index (OSTI). The red dotted line is the line of zero.

To study how membrane potential (V_m_)-to-spike transformation in SGS neurons impact their feature selectivity, we carried out *in vivo* intracellular whole-cell recording in urethane-anesthetized mice. We recorded SGS neurons under current clamp to reveal their spiking and the underlying V_m_ changes in response to sweeping bars in different directions (Figures 1A–D). As described in previous studies (Wang et al., 2010; Inayat et al., 2015), SGS neurons displayed diverse feature selectivity. To moving bars, some cells showed orientation selectivity (OS; Figure 1C), and others showed DS (Figure 1D). We separately analyzed the recorded cells’ V_m_ and spike responses and examined their tuning properties (see “Materials and Methods” section for details; e.g., Figures 1E–H). Using the normalized vector sum as an index (see “Materials and Methods” section for details), which we refer to as gDSI and gOSI, we quantified the degree of DS and OS of spiking and V_m_ responses, respectively, of the recorded SGS neurons. As expected from the thresholding effect (Carandini and Ferster, 2000), the gDSI or gOSI of spiking responses were greater than those of V_m_ responses for individual cells (e.g., Figures 1G,H) and across the population (Figures 2A–D).

We then quantified the transformation of direction/orientation selectivity from V_m_ to spiking responses by calculating a DS transformation index (DSTI = gDSI-Spike − gDSI-V_m_) and an OS transformation index (OSTI = gOSI-Spike − gOSI-V_m_). Most of OSTI and DSTI were positive, reflecting the sharpening of OS and DS from SGS neurons’ input (V_m_) to output (spikes). Furthermore, the degree of this sharpening was highly variable among the recorded cells, with OSTI ranging from −0.04 to 0.31, and DSTI from −0.02 to 0.27, respectively. No correlation between DSTI and gDSI-V_m_ or OSTI and gOSI-V_m_ were found in the recorded cells (Figures 2E,F), suggesting that the feature selectivity of input itself does not determine the degree of its transformation from input to output.

### Receptive Field Size Constrains V_m_-to-Spike Transformation of Feature Selectivity

**Figure 3:**
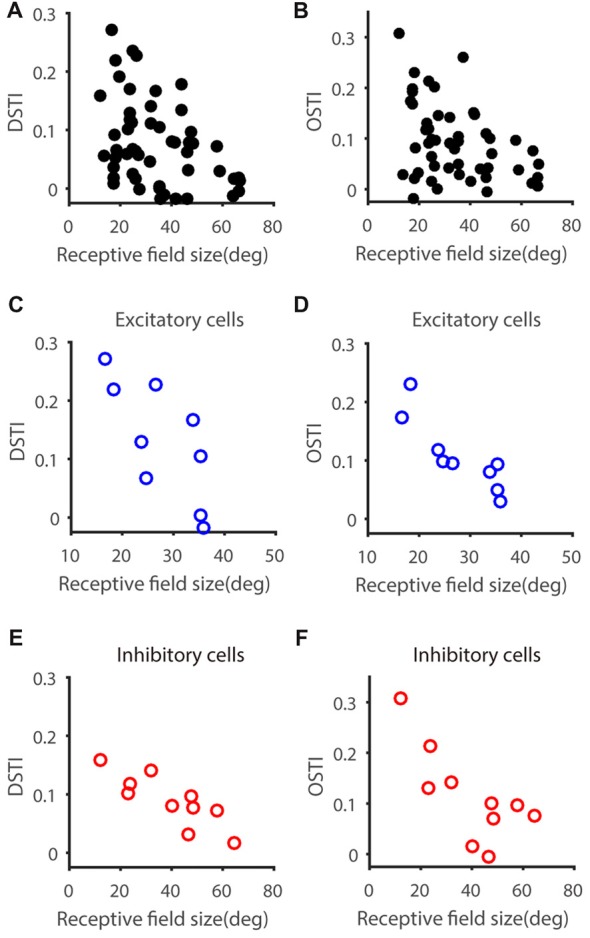
Relationship between feature transformation and receptive field (RF) size. **(A,B)** Scatter plot of RF size vs. V_m_-to-spike-rate DSTI **(A)** and OSTI **(B)**, indicating significant correlation (*n* = 52 cells from 41 mice, *r* = −0.40, *P* < 0.01 for panel **A**, *r* = −0.37, *P* < 0.01 for panel **B**). **(C,D)** Scatter plot of RF size vs. DSTI **(C)** and OSTI **(D)** of identified excitatory cells (*n* = 9; *r* = −0.74, *P* < 0.05 for panel **C**, *r* = −0.87, *P* < 0.01 for panel **D**). **(E,F)** Scatter plot of RF size vs. DSTI **(E)** and OSTI **(F)** of identified inhibitory cells (*n* = 10; *r* = −0.84, *P* < 0.01 for panel **E**, *r* = −0.73, *P* < 0.05 for panel **F**).

We next asked whether other characteristic of the input (as reflected by V_m_) constrains the feature selectivity transformation from V_m_ to spikes. One possibility is that the RF size of input may have an impact on the DS or OS transformation from V_m_ to spiking responses, since cells with big or small RF may take different roles in visual processing. To explore this possibility, we measured the RF size of V_m_ of each recorded neuron (see “Materials and Methods” section), which is larger than that of spiking responses and reflect the full range of visual field to which the recorded neurons respond, including subthreshold responses. Neither gDSI or gOSI itself was significantly correlated with RF size (gDSI-Spike, *r* = −0.26, *P* = 0.07; gDSI-V_m_, *r* = −0.10, *P* = 0.48; gOSI-Spike, *r* = −0.25, *P* = 0.07; gOSI-V_m_, *r* = 0.04, *P* = 0.76), but the increase of gDSI and gOSI (i.e., the “transformation index”, DSTI and OSTI) were negatively correlated with the RF size in the recorded cells (DSTI: *r* = −0.40, *P* < 0.01, Figure 3A; OSTI: *r* = −0.38, *P* < 0.01, Figure 3B; *n* = 52 cells from 41 mice). Furthermore, using transgenic mice that expressed Channelrhodopsin-2 (ChR2) in GABAergic inhibitory neurons, we were able to identify whether the recorded cells were excitatory or inhibitory by their responses to LED photoactivation (Shi et al., 2017). The correlation between RF size and the transformation index was seen for both cell types and for both DSTI (*r* = −0.74, *P* < 0.05 for excitatory cells, *n* = 9 cells from 8 mice, Figure 3C; *r* = −0.84, *P* < 0.01 for inhibitory cells, *n* = 10 cells from 10 mice, Figure 3E) and OSTI (*r* = −0.87, *P* < 0.01 for excitatory cells, Figure 3D; *r* = −0.72, *P* < 0.05 for inhibitory cells, Figure 3F).

### V_m_-Spike Relationship in SGS Neurons

**Figure 4:**
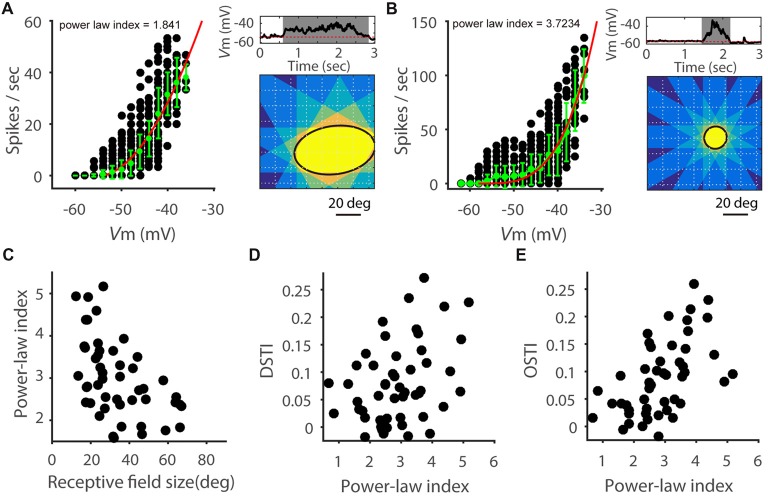
Power-law relationship of V_m_-to-spike transformation.** (A,B)** Relationship between V_m_ and spike rate in two example SGS neurons. On the left of each panel, each black point represents the spike rate and corresponding V_m_ response (grouped in 2 mV intervals) for one 50 ms epoch of a trial-averaged response. Green points and error bars are means and STDs of all the black points in the corresponding V_m_ range. Red curve is a fit of the green points to a power-law function. Right up: trial-averaged V_m_ response with the widest time window to the sweeping bar. Gray shadow indicates the response time window. Red dotted line indicates the resting V_m_. Right bottom: ellipse fitted RF (black solid line) measured with superimposed time windows of responses to 12 directions of moving bars. Note that the power-law index of the cell in panel **(A)** is smaller than that of **(B)**, and its RF size is larger than that of the cell in panel **(B)**. **(C)** Scatter plot of power-law index vs. RF size, indicating negative correlation (*n* = 52 cells from 41 mice, *r* = −0.45, *P* < 0.001). **(D)** Scatter plot of DSTI vs. power law index (*r* = 0.38, *P* < 0.01). **(E)** Scatter plot of OSTI vs. power-law index (*r* = 0.62, *P* < 0.0001).

We next sought insights on what biophysical properties might contribute to the observed correlation between feature selectivity transformation and RF size. Previous studies in visual cortex have shown that V_m_-spike transformation follows a power-law nonlinearity (Priebe and Ferster, 2008) and neurons with higher power-law index tended to have a greater OSI increase from V_m_ to spike rate than neurons with lower index (Tan et al., 2011). We thus examined this factor by fitting power-law functions to the data of spike rate vs. V_m_ (see “Materials and Methods” section). As in previous studies, we used the exponent of the fitted function, referred to as power-law index (Priebe and Ferster, 2008; Tan et al., 2011), to quantify the V_m_-spike transformation (Figures 4A,B). No significant difference in the power law index was seen between excitatory (3.38 ± 0.33, *n* = 9) and inhibitory cells (3.01 ± 0.41, *n* = 10; *t* = 0.70; *P* = 0.49). Interestingly, the power-law index was indeed negatively correlated with the RF size (Figure 4C, *r* = −0.45, *P* < 0.001), where cells with larger RFs tended to have smaller exponents (e.g., Figure 4A) and smaller RFs larger exponents (e.g., Figure 4B). Consequently, both DSTI (Figure 4D, *r* = 0.38, *P* < 0.01) and OSTI (Figure 4E, *r* = 0.61, *P* < 0.0001) were positively correlated with the power-law index.

**Figure 5:**
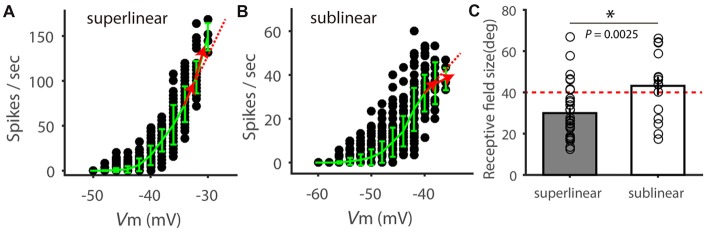
Superlinear and sublinear V_m_-spike transformation in SGS neurons. **(A)** Relationship between V_m_ and spike rate in an example SGS neuron. Red arrows show the instant change of spike rate with V_m_ increasing, which is higher than that at lower V_m_ (dotted red line), indicating superlinear change of spike rate with V_m_. **(B)** Relationship between V_m_ and spike rate in another example SGS neuron, indicating sublinear change of spike rate at the more depolarized V_m_. **(C)** Comparison of RF size between SGS neurons with superlinear and sublinear V_m_-spike-transformation. There is significant difference between these two groups (*t* = 3.18, *P* < 0.01). Note that most “superlinear” cells (80.0%, 28/35) had RF size smaller than 40 degrees (red dotted line), and most “sublinear” cells (64.7%, 11/17) had RF size larger than 40 degrees. Error bars represent STD in **(A,B)** and SEM in **(C)**. * represents *P* < 0.01.

In performing this analysis, we noted that not all V_m_-spike relationship could be best fitted by the power-law nonlinearity, possibly due to sublinear effects at more depolarized V_m_ (Zhao et al., 2013b). We thus carried out additional analysis and separated the recorded cells into “superlinear” and “sublinear” cells based on their behavior at the most depolarized membrane potentials. Specifically, we calculated spike rate change in the two most depolarized V_m_ ranges (shown as red arrows in Figures 5A,B). Cells with increasing rate change with V_m_ were considered “superlinear cells” (e.g., Figure 5A) and cells with decreasing rate changes were “sublinear cells” (e.g., Figure 5B). Overall, the “superlinear cells” had smaller RFs than the “sublinear cells” (Figure 5C). The above analyses thus suggest that the V_m_-spike relationship in SGS cells is correlated with their RF size, and likely contributes to the RF-dependent feature selectivity transformation.

### Spike Initiation in SGS Neurons

**Figure 6:**
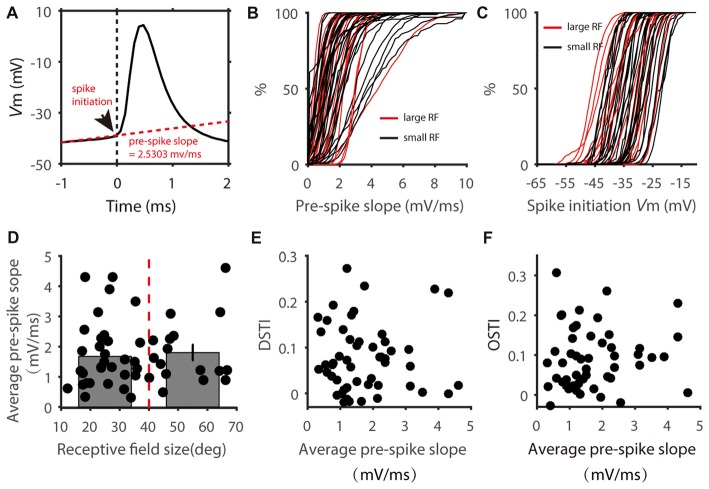
Distribution of pre-spike V_m_ slopes.** (A)** Calculation of pre-spike V_m_ slope. Arrow head shows the point of spike initiation. The slope of the red dotted line shows the mean pre-spike V_m_ slope during the 1 ms window before spike onset. **(B)** Cumulative distributions of pre-spike V_m_ slope of cells with small (black) and large (red) RFs. Each line represent all spikes from one cell.** (C)** Cumulative distributions of spike initiating V_m_ of the two groups of cells. **(D)** Scatter plot of average pre-spike slope vs. RF size. The average pre-spike slopes of two groups divided at 40 degrees of RF size (red dotted line) showed no significant difference (*t* = 0.37, *P* = 0.71).** (E,F)** Scatter plot of DSTI **(E)** and OSTI **(F)** vs. average pre-spike slope.

The spike threshold is highly variable *in vivo* (Azouz and Gray, 2000; Fontaine et al., 2014), which could be stimulus dependent and affect feature selectivity in cortical neurons (Wilent and Contreras, 2005). Moreover, spike initiation not only depends on the absolute V_m_ level but also on other features such as the rate of V_m_ change (Wester and Contreras, 2013). We thus calculated the slope of the V_m_ during the 1 ms window before each spike (Figure 6A) for individual neurons. For each cell, we plotted the distribution of this “pre-spike slope” (Figure 6B) and saw no obvious segregation of these distributions according to RF size. Similarly, distributions of spike initiating V_m_ also showed no difference between the two groups (Figure 6C). We then averaged the pre-spike slopes of all the spikes for each cell. Again, no correlation was observed between the average pre-spike slope and RF size; and the pre-spike slope in the cells with small RF was not different from that of the cells with large RF (Figure 6D). Accordingly, the DS and OS transformation index (DSTI and OSTI) did not show correlation with pre-spike slope in the recorded SGS cells (Figures 6E,F). Because small pre-spike slope is often associated with dendritic spikes which has been found in wide-field SGS cells (Gale and Murphy, 2016), our results suggest that it is unlikely that dendritic processing has much effect on feature selectivity transformation from V_m_ to spikes in mouse SGS neurons.

**Figure 7:**
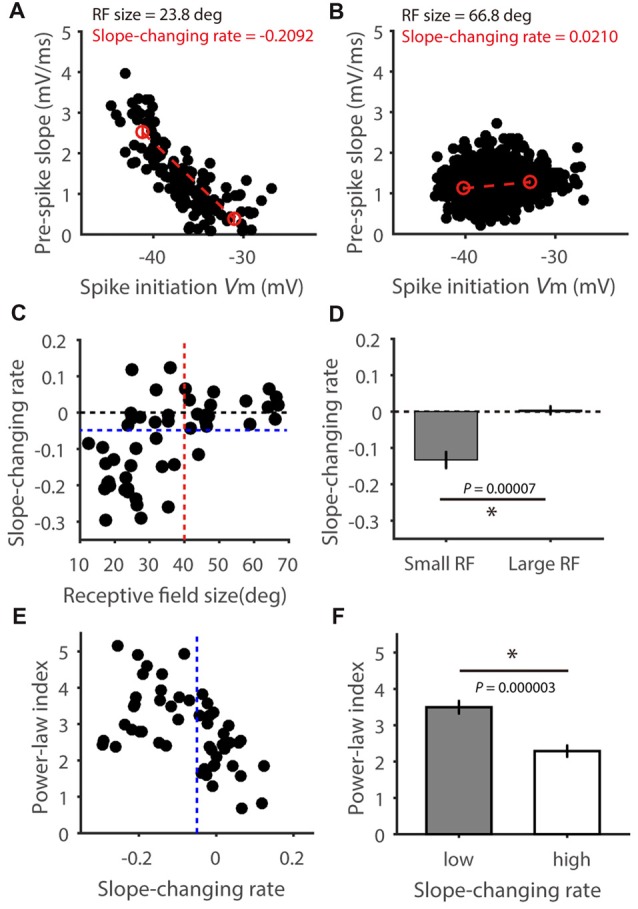
The relationship between pre-spike V_m_ slope and spike-initiating V_m_. **(A,B)** Relationship between pre-spike V_m_ slope and spike initiating V_m_ level in two example cells, one with small RF **(A)** and the other large **(B)**. Red circles indicate average pre-spike slopes for all spikes in the 0%–25% and 75%–100% spike initiating V_m_ range. Red dotted lines indicate the pre-spike slope changing rates. **(C)** Scatter plot of the “slope-changing rate” vs. RF size, indicating positive correlation between them (*r* = 0.60, *P* < 0.0001). Note that most of the cells (31/34, 91.2%) with RF smaller than 40 degrees (red dotted line) had values smaller than zero, and most of the cells with larger RFs (17/18, 94.4%) had values around zero and larger than −0.5 (blue dotted line). **(D)** The “slope-changing rate” is significantly different between the two groups of cells (*t* = 4.35, *P* < 0.0001). **(E)** Scatter plot of power-law index vs. slope changing rate, indicating negative correlation between them (*r* = 0.49 *P* = 0.0002), and **(F)** the means were significantly different between cells with high and low changing rate (*t* = 5.29, *P* < 0.0001). Error bars represent SEM. * represents *P* < 0.0001.

Finally, we analyzed the relationship between pre-spike slope and spike-initiating V_m_ for each cell. In some cells, a negative correlation was seen between the pre-spike slope and V_m_ threshold (e.g., Figure 7A), indicating a faster V_m_ rise is needed for spike initiation at more hyperpolarized V_m_. In other cells, their relationship is rather flat (e.g., Figure 7B). To quantify this relationship, we compared the average pre-spike slopes between the 0%–25% and 75%–100% of the observed V_m_ range for spike initiation (red circles in Figures 7A,B), and normalized their difference by the corresponding V_m_ difference. Notably, this “slope-changing rate” was well correlated with the RF size (*r* = 0.60, *P* < 0.0001, Figure 7C). Most cells with smaller RFs had negative values (Figure 7A for an example cell, Figures 7C,D for the population), while cells with larger RFs had values around zero (Figure 7B for an example cell, Figures 7C,D for the population; *t* = 4.35, *P* < 0.0001 for comparison between the two groups). In other words, for cells with smaller RFs, their spike initiation would become “easier” at more depolarized V_m_ because smaller V_m_ rise rate is needed, which would then contribute to their superlinear behavior in V_m_-spike transformation (Figure 5C) and higher power-law index than bigger RF neurons (Figures 7E,F). Overall, these results suggest that RF-size-dependent spike initiation is another factor contributing to feature selectivity transformation from V_m_ to spikes.

## Discussion

Feature selectivity is a fundamental property that allows neurons in the sensory systems to extract and encode external information. The circuit and synaptic mechanisms underlying feature selectivity have been extensively studied in the visual system, where neurons are tuned to specific stimulus orientation or moving direction. These studies have revealed precise neural circuits that give rise to orientation and direction selectivity in different visual structures. For example, direction selective ganglion cells (DSGCs) in the retina acquire their selectivity from the intricate spatial and temporal interactions between synaptic excitation and inhibition (Wei and Feller, 2011; Vaney et al., 2012). Similarly, orientation selectivity in the visual cortex arises from the excitatory thalamic inputs that are precisely aligned in their spatial RFs (Ferster and Miller, 2000; Priebe and Ferster, 2008; Priebe, 2016). On the other hand, we have recently discovered that SGS neurons in the SC inherit their direction selectivity from the retina by precisely converging inputs of similarly-tuned DSGCs (Shi et al., 2017).

In addition to the precise circuit connectivity, the transformation from synaptic input to spike output is another key mechanism in processing feature selectivity. Here, we performed *in vivo* whole cell recording of mouse SGS neurons to study factors important for orientation and direction selectivity transformation from V_m_ to spikes. We found that the selectivity increased from the V_m_ to spike responses, and the degree of this increase was highly variable among different neurons. Interestingly, the variability was not random, but correlated with the size of the neurons’ RFs. Our subsequent analyses indicate that the power-law relationship between V_m_ and spike rate and the relationship between V_m_ dynamics and spike initiation, but not dendritic processing, are RF size dependent and likely contribute to the observed input-output transformation of feature selectivity.

The power-law index, i.e., the exponent of the fitted power-law function, has been used to estimate the degree of V_m_-to-spike transformation in previous studies (Finn et al., 2007; Priebe and Ferster, 2008, 2012; Tan et al., 2011). Similar as in visual cortex (Tan et al., 2011), our studies showed that the power-law index is positively correlated with the level of increase of feature selectivity from V_m_ to spikes. In other words, the higher the index, the greater degree of the V_m_-to-spike sharpening of orientation/direction selectivity will be. Previous studies in cat V1 demonstrated that the power-law relationship between V_m_ and spike rate could be explained by trial-to-trial response variability (Hansel and van Vreeswijk, 2002; Miller and Troyer, 2002; Carandini, 2004; Priebe et al., 2004). Our finding that the power-law index is correlated with RF size, which would not affect response variability, suggests that additional mechanisms may be also involved. One possible factor is the relationship between spike initiating V_m_ and the required depolarizing rate, which we found to be correlated with RF size in mouse SGS neurons. Cells with smaller RFs display a negative correlation, where spike initiation becomes “easier” at more depolarized V_m_ because smaller V_m_ rise rate is needed. These cells would likely show higher power-law index and superlinear behavior in V_m_-spike transformation. In contrast, cells with larger RFs require similar depolarization rate for spike initiation at different V_m_, thus displaying smaller power-law index than smaller RF cells.

What causes the differences in the relationship between V_m_ and spike rate and between V_m_ dynamics and spike initiation among cells with different RF sizes is still unknown. One possibility is that cells with different RFs may represent distinct cell types. We were not able to distinguish the recorded cells into subtypes because of the limited dataset in reconstructed morphology. But it was shown that narrow field (NF) vertical cells, wide field (WF) vertical and stellate cells are the three types of excitatory cells and they show different RF size (Gale and Murphy, 2014). It is possible that these cells have different biophysical features in their membrane properties that underlie their difference in V_m_-to-spike transformation. On the other hand, horizontal cells represent possibly the only inhibitory cell type in the mouse SGS (Gale and Murphy, 2014), and our optogenetic experiments demonstrate that they also display RF-size-dependent V_m_-to-spike transformation. This result suggests that additional factors within a cell type could contribute to the observed difference as well.

Finally, the RF-dependent differences in V_m_-to-spike transformation may have functional implications. For small visual objects such as a prey that is best responded to by cells with small RFs, a higher input-output transformation could augment the saliency of the objects. For large visual stimuli such as a moving background, a lower transformation could maintain the wider tuning of the input, potentially helping keep the fidelity of the signal. These speculations may be testable one day under more natural conditions with ethologically-relevant visual stimulation. Our current study thus provides the basic information for future studies that will attempt to link sensory input to behavioral output.

## Author Contributions

XS and JC designed the experiments. XS performed *in vivo* whole-cell recording experiments and analyzed the data. YJ performed histology. JC guided data analysis and oversaw the project. All authors discussed the results and wrote the manuscript.

## Conflict of Interest Statement

The authors declare that the research was conducted in the absence of any commercial or financial relationships that could be construed as a potential conflict of interest.
